# De-Bruijn graph partitioning for scalable and accurate DNA storage processing

**DOI:** 10.1093/bioinformatics/btaf618

**Published:** 2025-11-09

**Authors:** Florestan De Moor, Olivier Boullé, Dominique Lavenier

**Affiliations:** IRISA, CNRS, Rennes, 35042, France; IRISA, CNRS, Rennes, 35042, France; IRISA, CNRS, Rennes, 35042, France

## Abstract

**Motivation:**

DNA-based data storage offers a compelling solution for long-term, high-density archiving. In this framework, accurately reconstructing high-quality encoded sequences after sequencing is critical, as it directly impacts the design of error-correcting codes optimized for DNA storage. Furthermore, efficient and scalable processing is essential to manage the large volumes of data expected in such applications.

**Results:**

We introduce a novel method based on de-Bruijn graph partitioning, enabling fast and accurate processing of sequencing data regardless of the underlying sequencing technology and without requiring prior knowledge of the information encoded in the oligonucleotides. Evaluated on both synthetic and real datasets, the method achieves excellent precision and recall. It is implemented in C++ within the software ConCluD and optimized for multi-core servers. Our experiments show that a dataset of 89 million reads, corresponding to a 10 GB fasta file, can be fully processed in less than a minute on a standard 32-cores server.

**Availability and implementation:**

The ConCluD software and the scripts to reproduce the experiments from this paper are available at https://gitlab.inria.fr/pim/org.pim.dnarxiv under the GNU AGPLv3 licence. An archival snapshot of the repository is also provided at https://doi.org/10.5281/zenodo.17160067.

## 1 Introduction

The world is experiencing an exponential surge in digital information, posing significant challenges for archival storage. Existing technologies consume large amounts of energy, occupy substantial physical space, incur high infrastructure costs, and require extensive maintenance to ensure long-term data preservation. Advances in molecular synthesis and the rise of efficient sequencing technologies have opened the possibility of storing data in DNA molecules. This approach is gaining interest as a potential solution, leading to increased effort to overcome the current technological barriers hindering its widespread adoption (Church *et al.* 2012; Ceze *et al.* 2019; Meiser *et al.* 2020).

**Figure 1.**
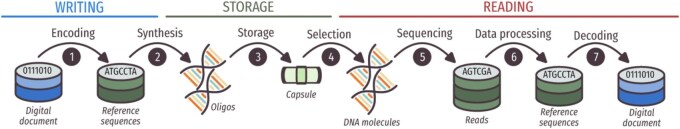
Key steps of a DNA storage system. This paper focuses on step 6: processing sequencing reads.

DNA-based data storage follows several key steps, as illustrated in Fig. 1. First, binary files are encoded into a DNA sequence representation. Then, DNA molecules are synthesized based on this encoded data and stored in a protected environment. To retrieve the information, the process begins by selecting the DNA molecules that correspond to the desired files. These molecules are then sequenced, and the resulting data are transmitted to the processing and decoding stages to reconstruct the original files.

Current synthesis processes can only produce short oligonucleotides with a maximum length of about 300 nucleotides. As a result, files must be divided into smaller fragments, each of which is indexed to allow the reconstruction of the original information. For example, a video file would be fragmented into hundreds of millions of oligonucleotides.

In a single storage unit, thousands of different documents can co-exist. To identify them, each file corresponds to a unique nucleotide sequence that acts like a tag. This tag not only helps distinguish files, but can also be used to biochemically select the oligonucleotides associated with a specific file. In this case, the tag can be designed as a pair of primers. This enables targeted amplification of the molecules using PCR, followed by a sequencing of the PCR product.

However, the sequencing output cannot be sent directly to the decoding stage. The data must first be processed to eliminate redundancy introduced during sequencing and to reconstruct the initial set of sequences that represent the file as accurately as possible. The first processing step involves clustering the reads to group similar sequences together. In the second step, a consensus sequence is established within each group, allowing for the correction of potential errors. This process ensures the production of high-quality sequences, which can then be transmitted to the decoder.

Since sequencing is a stochastic process, it is highly likely that not all sequences will be successfully reconstructed: some of those provided to the decoder may still contain errors. To address this issue, the encoding process incorporates error-correcting codes. These codes enable the reconstruction of the complete information from only a subset of recovered sequences while also correcting residual errors, ensuring data integrity (Erlich and Zielinski 2017; Marinelli *et al.* 2024).

The work presented here focuses on processing sequencing data with the goal of delivering as many correct sequences as possible to the decoder. Similar work for genomic purpose has already been carried out, mainly in bioinformatics and metagenomics, but they mainly focuses on DNA clustering algorithms. Methods such as UCLUST (Edgar 2010), CD-HIT (Fu *et al.* 2012), and DNACLUST (Ghodsi *et al.* 2011) use greedy algorithms for sequence similarity, while MeShClust (James *et al.* 2018) and MetaDEC (Bao *et al.* 2022) use machine learning approaches. Graph-based techniques, such as MMseqs2 (Steinegger and Söding 2018), offer further strategies. However, a common limitation of these algorithms is their reliance on a fixed sequence identity threshold, which can be difficult to determine, particularly in DNA storage systems. Unlike genomic clustering, DNA data storage presents unique constraints due to shorter DNA strands (100–300 nucleotides). Although algorithms such as ALFATClust (Chiu and Ong 2022) and SEED (Bao *et al.* 2011) attempt to dynamically adjust clustering parameters, they remain optimized for metagenomic data. Recent advances in large-scale DNA clustering for storage applications include the work of Rashtchian *et al.* (2017) and GradHC (Ben Shabat *et al.* 2024), both of which are capable of clustering billions of reads and producing highly reliable clustering results. The output of these programs are clusters containing a small number of reads, which are then used to build a consensus sequence.

Our approach is distinct and aims to merge the clustering and consensus steps into a single process. To achieve this, we draw inspiration from bioinformatics techniques used in genome assembly, particularly the use of de-Bruijn graphs (Pevzner *et al.* 2001; Zerbino and Birney 2008). This method has already been explored by Song *et al.* (2022) and has proven to be particularly robust in handling DNA breaks, rearrangements, and indels. However, as the size of the encoded files increases, the construction of large de-Bruijn graphs can lead to excessive computation times.

Our method reduces algorithmic complexity by hierarchically dividing the initial set of reads into smaller subsets of predefined size. Each subset is expected to contain a portion of the original sequences along with their noisy copies. A de-Bruijn graph is then constructed for each subset. Given the known length (N) of the target sequences and the nucleotide sequences that mark their start and end (the primers), the sequences to be reconstructed correspond to all paths of length N between two k-mers derived from the start and end primers. The advantages of this method are: (a) the hierarchical partitioning is extremely fast; (b) constructing smaller de-Bruijn graphs is also efficient, as it relies on reduced k-mer sizes, enabling optimized k-mer counting techniques; (c) parallelization on multicore architectures is straightforward since there are no dependencies between the de-Bruijn graphs.

## 2 Method

In the context of DNA storage, reference sequences consist of three concatenated parts: a start primer, a payload, and an end primer. The payload carries the encoded data, along with indexing information for document reconstruction, optional checksums, and error correction data. To retrieve a document, a PCR is first performed using the specific primer pair associated with it, followed by a sequencing process. The resulting dataset may contain billions of reads. We make the assumption that each read includes a complete sequence, encompassing both primers.

To recover the initial set of sequences, a straightforward strategy is to construct a de-Bruijn graph using all these sequences. Since they all share the same start and end primers, we could enumerate all paths in the graph that begin with a k-mer extracted from the start primer and end with a k-mer from the end primer. Each path represents a possible sequence. Paths that do not have the correct length are discarded. In practice, this solution works well and remains relatively fast for graphs of reasonable size. However, as the number of sequences increases, the computation time grows exponentially. Constructing a de-Bruijn graph involves counting k-mers, which is indeed a computationally intensive task. Also, identical k-mers in different sequences increase the number of possible paths, drastically raising the complexity of the problem.

**Figure 2.**
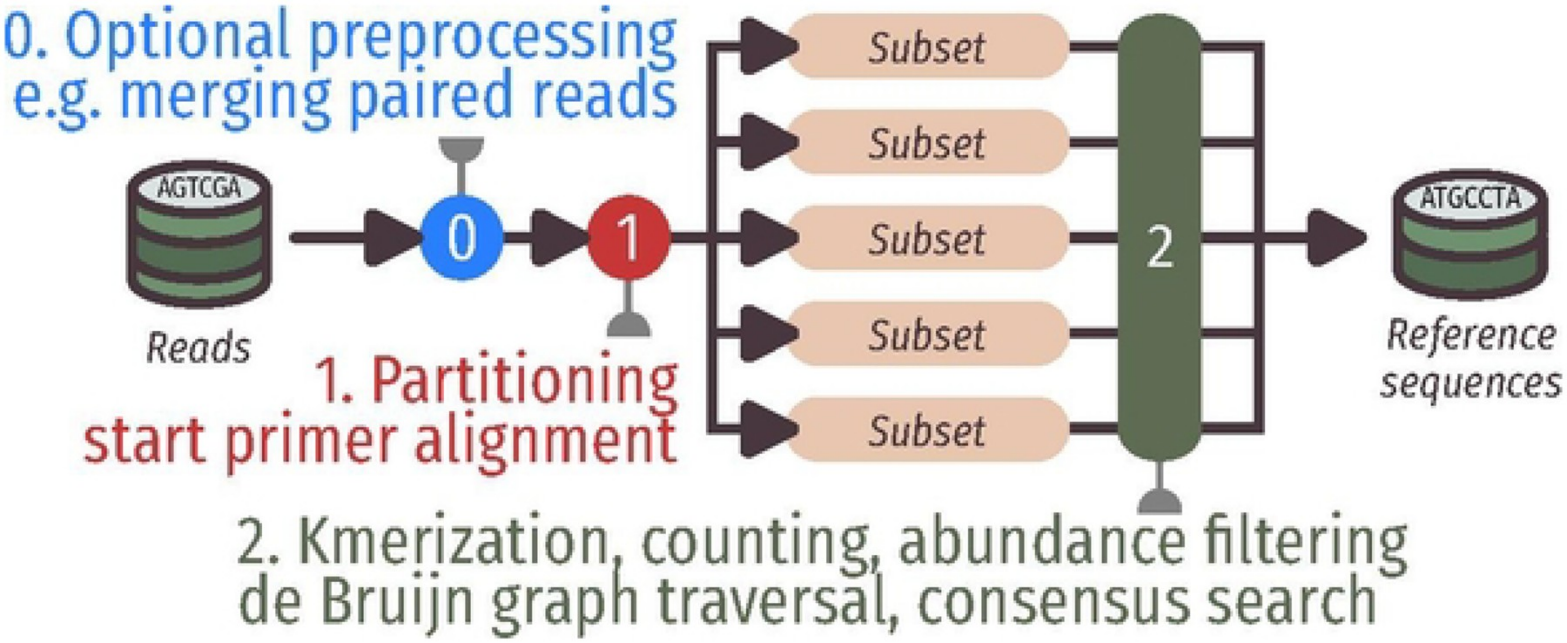
Overview of our method. Step 1 aligns the start primers and partitions all reads. Step 2 builds de-Bruijn graphs and traverses them to find consensus sequences of the expected length.

To mitigate this complexity, our method first partitions the dataset into multiple subsets of approximately equal size. Each subset, and it is of the most importance, contains all instances of a given original sequence. The problem then consists of searching for paths in many independent graphs of reduced size, which can be done as mentioned previously. The algorithm represented in Fig. 2 can be abstracted as follows:

1. Partition the dataset into subsets S of roughly equal size
2. **for** each subset *s* in S  **do** 
3.   Create a de-Bruijn graph G from *s*
4.   Generate consensus sequences from G
5. end for

To accelerate computations in a multithreaded environment, line 1 benefits from intra-read parallelism, while lines 2–5 allow for intra-subset parallelism.

### 2.1 Partitioning

The objective of this step is to distribute the entire set of reads into subsets so that all copies of the same sequence are present in the same subset. The first task is to identify the primer within the read, considering that the method is independent of the sequencing technology and that the primer is not necessarily located at the very beginning. To achieve this, we index k-mers in the read and perform a local alignment against the start primer (and its reverse complement) at promising positions. In our experiments, an elaborate approach taking gaps into account with the Smith-Waterman algorithm (Smith and Waterman 1981) led to negligible accuracy improvements over using a simple Hamming distance. We thus opted for the latter as it is less computationally expensive. Some reads may be assigned to no subset if no sufficiently good alignment is found, which is more likely to occur for reads with a high error rate. However, removing them is actually beneficial, as they could otherwise compromise the accuracy of the consensus search.

From the primer alignment position, the few bases immediately following the primer are used as a partitioning key. We start with four bases to make an initial batch of 256 subsets. Then for each, if it has more than a predefined number of reads *T*, we expand the key to 20 bases, sort all the reads in this subset by their key, and split the subset into files of at most *T* reads with a linear pass. This approach accounts for the unknown data distribution and ensures a more balanced load distribution, preventing some subsets from having an excessive number of reads while others remain nearly empty. When subsets get written to the disk, reads are trimmed to eliminate irrelevant parts: segments preceding the starting primer and segments after the expected length of the oligonucleotides encoding the original file. Reads are also reoriented identically.

Since large files are often indexed in several parts, each identified by a specific pair of primers, we adapt the method to accept several starting primers. The partitioning performs an initial split of reads based on which primer gives the best alignment score, before proceeding with the partitioning key.

This partitioning method is fast and efficient. Errors at unfortunate positions in the read will inevitably lead to assigning it to an inadequate subset. However, with current error rates and advances in sequencing technologies and base-calling algorithms, the number of misassigned reads remains fairly low, and does not disrupt processing in subsequent steps.

### 2.2 De-Bruijn graph building

De-Bruijn graphs are primarily built based on k-mer counting. The advantage of considering only small subsets (fewer than 2000 reads) is that the resulting graph will also be of limited size. The k-mer length can be optimally adjusted, enabling extremely simple and fast counting techniques that do not require complex data structures. In this case, a simple hash table is used and the values of the counters are capped to 255 to fit into 8-bit variables.

Finally, we discard the k-mers that appear less than a user-defined threshold. This filtering eliminates sequencing errors, but also k-mers from reads assigned to an improper subset.

### 2.3 Generating consensus sequences

The last stage consists of traversing the paths of each de-Bruijn graph to find consensus sequences.

The exploration begins at the node representing the starting k-mer, specifically the rightmost k-mer of the longest common prefix of all reads in the current subset. Then, the next possible four bases are considered recursively. If the traversal reaches the leftmost k-mer of a possible ending primer and the current payload has the exact expected size, the sequence is considered a consensus. If the traversal results in a sequence that exceeds the expected consensus length, the search on that particular path is terminated.

Since this exploration has exponential complexity, several techniques are employed to reduce processing time. First, the graph is progressively pruned by removing nodes with no outgoing edges. Second, when a path is terminated, the current sequence is analyzed for repeated k-mers. If repetitions are detected, one edge is removed from the graph to break the loop. This optimization is lossy, meaning that it may prevent some valid consensus sequences from being found. However, eliminating loops significantly accelerates the search process. As in any data transmission context, some sequences are expected to be missing, and efficient techniques such as error correction codes can be applied afterwards to address this issue.

## 3 Evaluation setup

### 3.1 Hardware

Hardware-wise, we use a server fitted with an Intel^®^ Xeon^®^ Silver 4215 CPU @ 2.5 GHz processor (Cascade Lake architecture, 16 hyper-threaded cores) and 256 GB of DDR4 @ 2.4 GHz RAM. The server operates on Debian 10.

### 3.2 Software

*ConCluD* (Consensus algorithm based on Clustering for DNA storage Decoding) is the name of the C++ software developed for the method described in the previous section. Unless stated otherwise, ConCluD is executed with 32 CPU threads and the following default parameter values: k-mer size = 15, k-mer abundance threshold = 3, and maximum number of reads in a subset = 2000.

*DBGPS* has been developed by Song *et al.* (2022) and also uses a de-Bruijn graph approach to reconstruct sequences after sequencing. A single graph is built from the whole read dataset. To reconstruct sequences, the oligonucleotides must be necessarily structured into three distinct fields: an index area, followed by the payload, and then a CRC field. The entire sequence is also framed by two primers. The C multithreaded version is used for comparison. It counts k-mers in parallel, but is sequential afterwards during the graph exploration.

*GradeHC* has been developed by Ben Shabat *et al.* (2024) and only generates clusters of reads. Since it does not implement the rest of the pipeline, it requires another software to then generate a single consensus sequence from each cluster.

### 3.3 Synthetic datasets

Table 1 summarizes the synthetic datasets we used for evaluating ConCluD. For each dataset, it gives the length of the oligonucleotides, the sequencing technology simulated, the number of primers, the numbers of oligonucleotides and reads. A detailed description of each of them, and the way they have been designed, can be found in the supplementary materials.

**Table 1.** List of synthetic datasets.

Name	Oligo len.	Seq. techno	# Pri.	# Oligos	# Reads
IM1-164ont2	164	ONT	2	31 294	455 871
IM1-164ill2	164	ILL	2	31 294	615 840
IM1-272ont2	272	ONT	2	35 090	533 488
IM10-164ont20	164	ONT	20	365 830	5 328 911
IM10-272ont20	272	ONT	20	350 690	5 331 761
IM10-272ont11	272	ONT	11	350 690	5 331 711
IM100-272ont20	272	ONT	20	3 511 400	53 385 593
SF-480ont2	480	ONT	2	12 258	184 918
SF-840ont2	840	ONT	2	6129	92 445
SF-1740ont2	1740	ONT	2	2724	41 077

The data are from high-resolution images. The payloads were constructed in compliance with the requirements for DNA storage (no large homopolymers, GC content around 50%, primers that do not hybridize in the middle of the payload, etc.). In addition, to ensure compatibility with the DBGPS dataset format, the same oligonucleotide structure was used for the IM1, IM10 and IM100 datasets, although ConCluD does not need any indexes to process the data. The ONT and Illumina sequencing datasets have been respectively generated with the PBSIM3 (Ono *et al.* 2022) and ART simulator tools (Huang *et al.* 2012). The coverage has been set to 30×.

The datasets IM1-xxx use a single image of 500 kB or 1.3 MB, and encode it with oligonucleotides of sizes respectively 164 nt or 272 nt, synthesized with ONT or Illumina read simulators. The datasets IM10-xxx use a single image of 5.8 MB or 13.5 MB, partition it into 10 parts, and encode each part with oligonucleotides of size respectively 164 nt or 272 nt. The parts in IM10-164ont20 and IM10-272ont20 are identified by different pairs of primers. On the contrary, the parts in IM10-272ont11 share the same start primer. IM100-272ont20 uses a single image of 134 MB, partitions it into 100 parts, and assigns to each part a unique pair of primers coming from a pool of 10 start primers and 10 end primers.

The datasets SF-xxx refer to the same image, but encoded with oligonucleotides of size 480 nt, 840 nt, or 1740 nt. The purpose of these three datasets is to test how ConCluD performs on large oligonucleotides. Since DBGPS primarily targets small oligonucleotides, these datasets will not be used for comparison.

### 3.4 Real datasets

To test and evaluate ConCluD on real data, we selected the real datasets listed in Table 2.

**Table 2.** List of real datasets.

Name	Ref.	Oligo len.	Seq. techno	# Oligos	# Reads
Park et al.	2025	198	ILL	18 000	0.36 M
Goldman et al.	2013	183	ILL	153 335	41.71 M
Odeuropa	2019	120	ONT	177 504	92.25 M
Dunhuang-0d	2022	200	ILL	210 000	16.37 M
Dunhuang-28d	2022	200	ILL	210 000	4.37 M
Dunhuang-56d	2022	200	ILL	210 000	1.53 M
HorrorMovie	2024	168	ONT	1 996 920	100.95 M

The Park dataset was designed using Fountain codes to explore how low-quality reads can be leveraged to reduce sequencing coverage requirements (Park *et al.* 2025). The Goldman dataset (Goldman *et al.* 2013) includes five files totaling 757 KB of data, in which text, images, and audio have been encoded. The Odeuropa dataset is provided by the OligoArchive European project (Appuswamy *et al.* 2019), and the Dunhuang-dX datasets originate from (Song *et al.* 2022) where ten digital pictures of Dunhuang murals have been encoded. Each Dunhuang datasets is based on the same initial sample but was subjected to accelerated aging for 0, 28, or 56 days to induce molecular degradation. Finally, the HorrorMovie dataset is derived from Malcolm Le Grice’s Horror Film 1, which was encoded into nearly two million nucleotides (Curham *et al.* 2024).

Datasets produced by Illumina paired-end technology cannot be used as is with ConCluD since the software requires both the start and end primers to be present in the same read. Fortunately, paired-end reads can be merged to reconstruct complete oligonucleotides. We used the NGmerge tool (Gaspar 2018) to this end. However, primer or adapter sequences could also be missing due to trimming during the base calling process, which fortunately reorients all sequences identically. To maintain compatibility with our approach, artificial start and end primers have been appended to restore the expected read structure.

### 3.5 Metrics

To evaluate the accuracy of our algorithm, we consider the precision and recall metrics.

Precision measures the amount of sequences returned by the program that are actually present in the reference. Achieving a good accuracy is important to ensure the reconstruction does not alter the original data. We compute the raw precision of the algorithm, independently of any checksum that might be embedded into the payload, and the final precision that allows to discard sequences after checksum verification.

On the other hand, recall measures the proportion of the reference that was found by the program. A bad recall hurts the reconstruction of information as some data blocks may be missing. We compute the raw recall of the program, independently of any error correction codes or other device that might be embedded into the payload to recover missing sequences.

Regarding time performance, we report the elapsed time of any execution.

## 4 Evaluation results

### 4.1 Precision and recall

**Figure 3.**
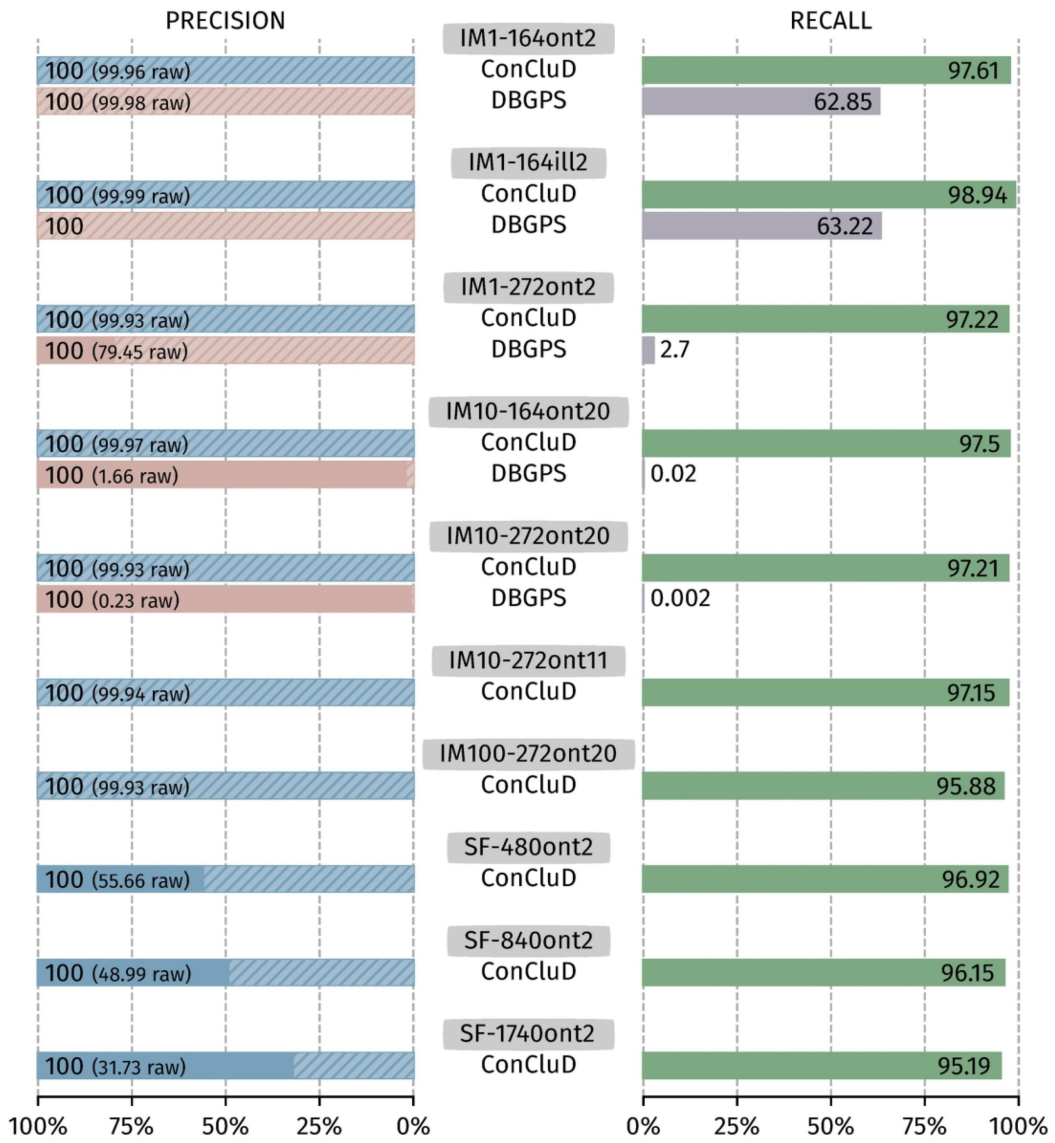
Precision and Recall on the synthetic datasets. Raw precision measures the amount of sequences returned by the software without looking at the CRC. Overall precision checks the CRC and is actually 100% on all datasets.

Figure 3 reports the precision and recall of ConCluD and DBGPS for all synthetic datasets. On small oligonucleotides (164 nt and 272 nt), ConCluD achieves excellent results in both precision and recall. On the other hand, DBGPS only produces satisfactory results for limited oligonucleotide sizes. For the SF datasets, DBGPS crashes with a stack smashing error. For dataset IM10-272ont11, the execution time is excessively long (24 minutes) and the output consensus sequences are erroneous. As for dataset IM100-272ont20, the process was killed due to memory overflow despite the 256 GB available. These poor results are due to the size of the de-Bruijn graph built by BDGPS, which is directly proportional to the number of reads, and thus of k-mers. The complexity of traversing such graph and reconstructing paths increases accordingly. While DBGPS removes loops in the graph to reduce complexity, this has an immediate impact: many paths supporting reference sequences are not explored. On the other hand, ConCluD integrates the same strategy but applies it to a multitude of small graphs, where only a few loops are removed, leading to marginal impact on the results.

As previously mentioned, the oligonucleotides were structured to be compatible with the DBGPS format. ConCluD relies on the nucleotides directly following the starting primer, which corresponds in this case to the added index. This could bias our strategy. Therefore, we tested our partitioning by using instead the position after the index, which corresponds to the beginning of the payload. The results remaining identical, we conclude no bias was introduced.

The last three datasets (SF-xxx) test the robustness of ConCluD against long oligonucleotides. A slight decrease in recall is observed when the length of the oligonucleotides increases. Regarding raw precision, the clear decrease is consistently compensated by the validity check.

This evaluation shows that achieving 100% recall is never possible, either because the synthesis did not produce all the expected oligonucleotides or because the random sequencing process failed to capture all the information. Error-correcting codes are thus critical to recover the missing information. Although this is not the focus of this work, we believe these results can help to quantify sequencing losses and adjust the parameters of error-correcting codes.

### 4.2 Execution time

**Figure 4.**
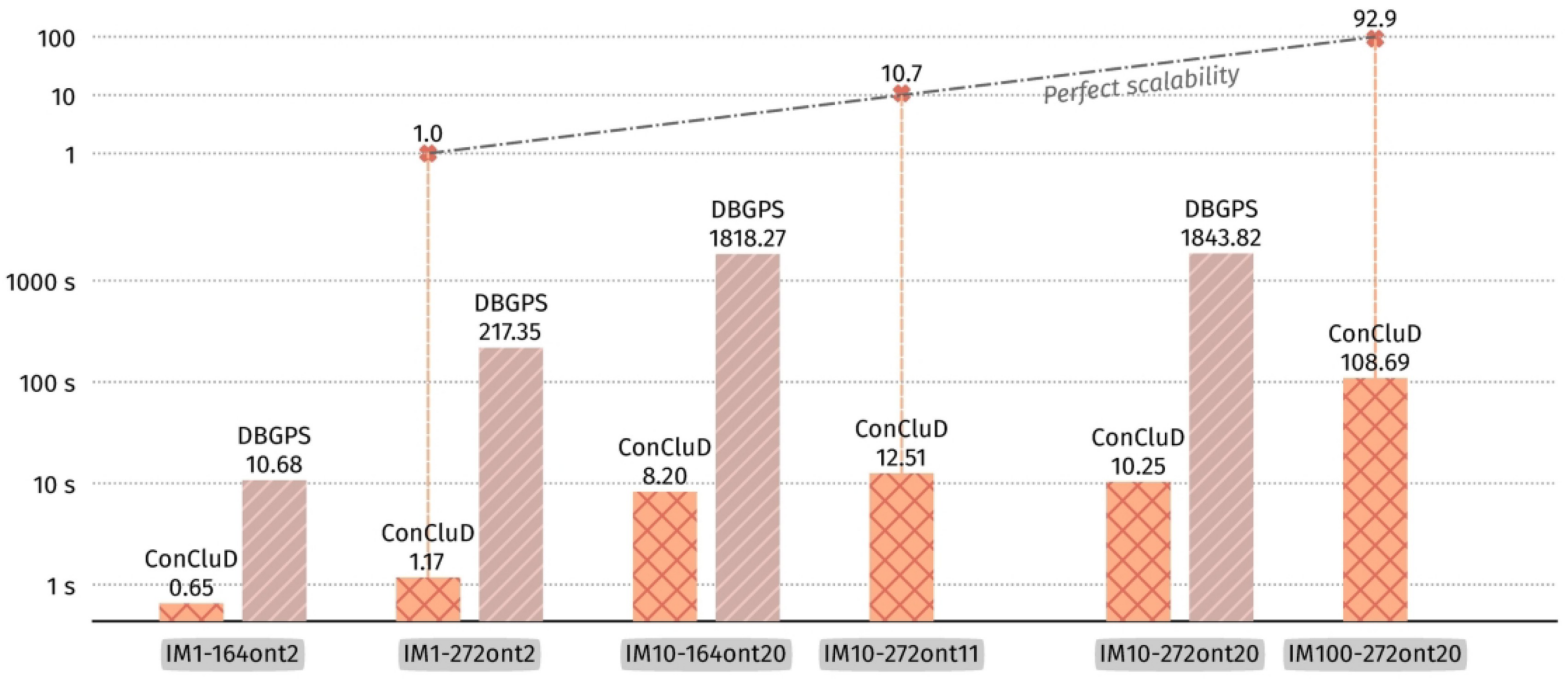
Execution time of ConCluD and DBGPS on synthetic datasets. For IM10-272ont11 and IM100-272ont20, DBGPS execution time is not reported as the software crashes or outputs no valid consensus.

Figure 4 shows the execution time for ConCluD and DBGPS on synthetic datasets. ConCluD clearly outperforms DBGPS. The top of the figure shows the relative execution time using the smallest dataset execution time as reference: we observe that the execution time of ConCluD increases almost linearly with the data size. The difference between IM10-272ont11 (with a common start primer for all images) and IM10-272ont22 (with different start and end primers for each image) favors the dataset using 20 primers. Having more starting primers decreases the likelihood of assigning a read to an irrelevant subset as the initial split based on finding the most appropriate primer clearly identifies different groups. This explains why the graph traversal stage is then more efficient and lead to a faster execution time overall.

GradHC only performs clustering and does not implement the full consensus pipeline. Therefore, we could not include precision and recall measures as it stands. Executing the software on our smallest dataset IM1-164ont2 already takes 40 minutes to output clusters, so it was not worth piping the clusters into another consensus software to produce comparable outputs. On this same dataset, ConCluD takes indeed less than a second to perform the full task, consensus included. Compared to ConCluD, the GradHC software is far too inefficient to be used in a practical pipeline.

### 4.3 Coverage

**Figure 5.**
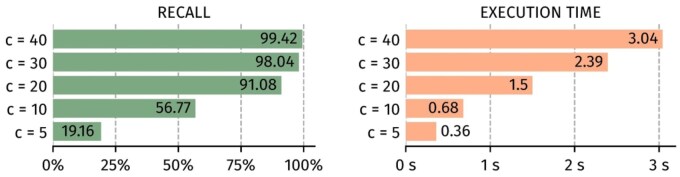
Impact of the sequencing coverage for recall and execution time.

In Fig. 5, we vary the subsampling of the Odeuropa dataset to evaluate coverage effects. We observe a satisfying recall when using a coverage of 30× or 40×. On the contrary, lower values significantly degrade the recall. Using a low coverage is cheaper and produces less reads, hence making the software faster, but it also leads to not having enough redundancy to reconstruct the original sequences. In the case of ConCluD, this means critical k-mers are missing in the de/¯Bruijn graph of each subset. It is thus important to be aware of this trade-off when setting up a DNA storage system.

### 4.4 Parameters impact

There are three main parameters that govern the operation of ConCluD: the size of the subsets in the partitioning phase, and the size of the k-mers and their solidity in the consensus phase. These parameters can influence the quality of the results (precision and recall) as well as the execution time. Experimental tests show that a subset size of 2000 reads, a k-mer size of 15 nt, and a low-abundance k-mer threshold of 3 provide good results and can be used as default parameters. In the following sections, the impact of these parameters is evaluated separately, with the others set to their default values.

**Figure 6.**
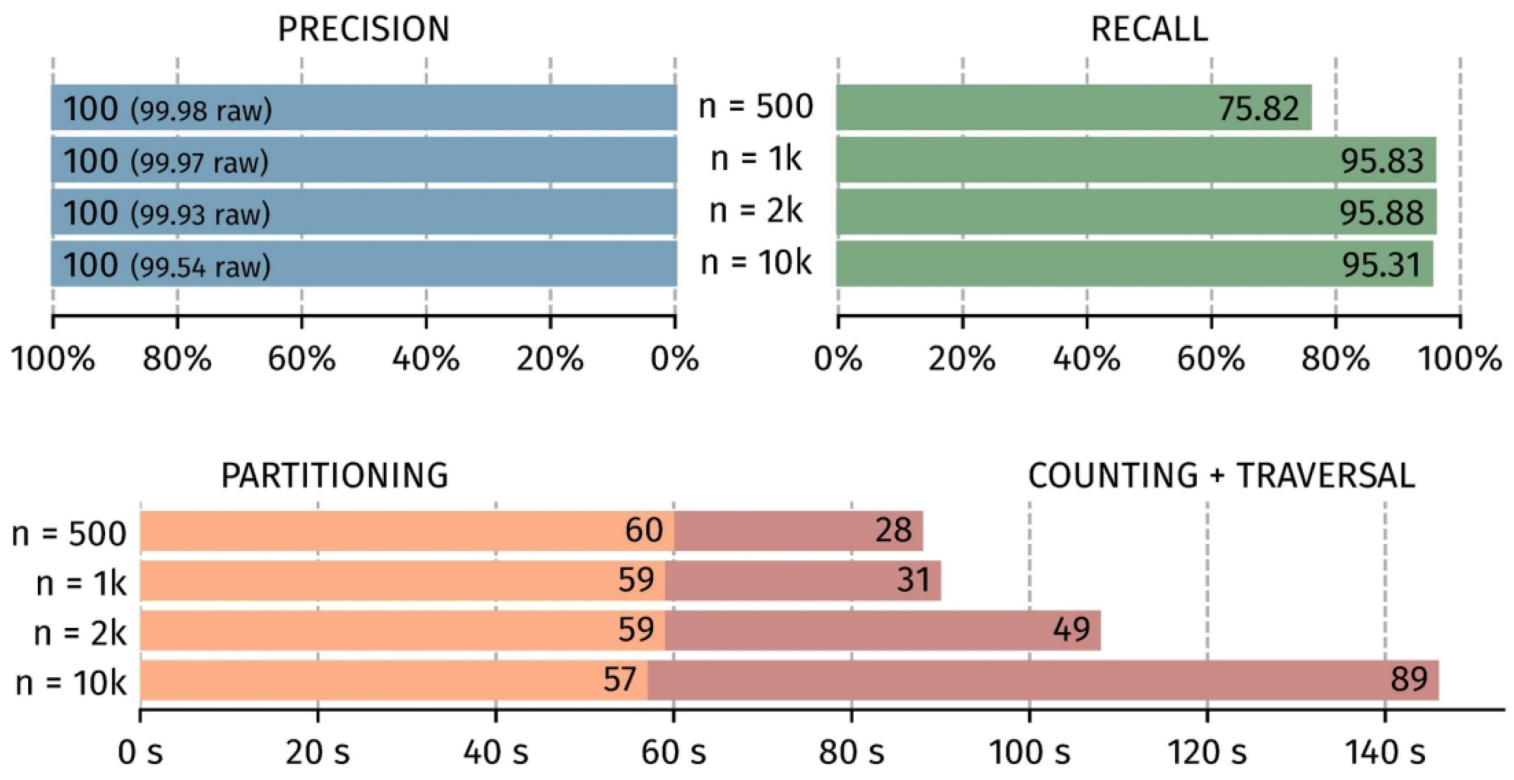
Impact of the subset size for precision, recall and execution time.

Figure 6 evaluates the impact of the subset maximum size using different values: 500, 1 k, 2 k, and 10 k reads. The results show that larger subsets reduce recall. This supports our strategy of managing multiple small de-Bruijn graphs rather than a single large one. However, subsets that are too small also decrease the recall. With larger subsets, the execution time of partitioning decreases slightly. However, the processing time for consensus increases more significantly. In the end, a maximum subset size of 2 k reads appears to be an optimal choice.

**Figure 7.**
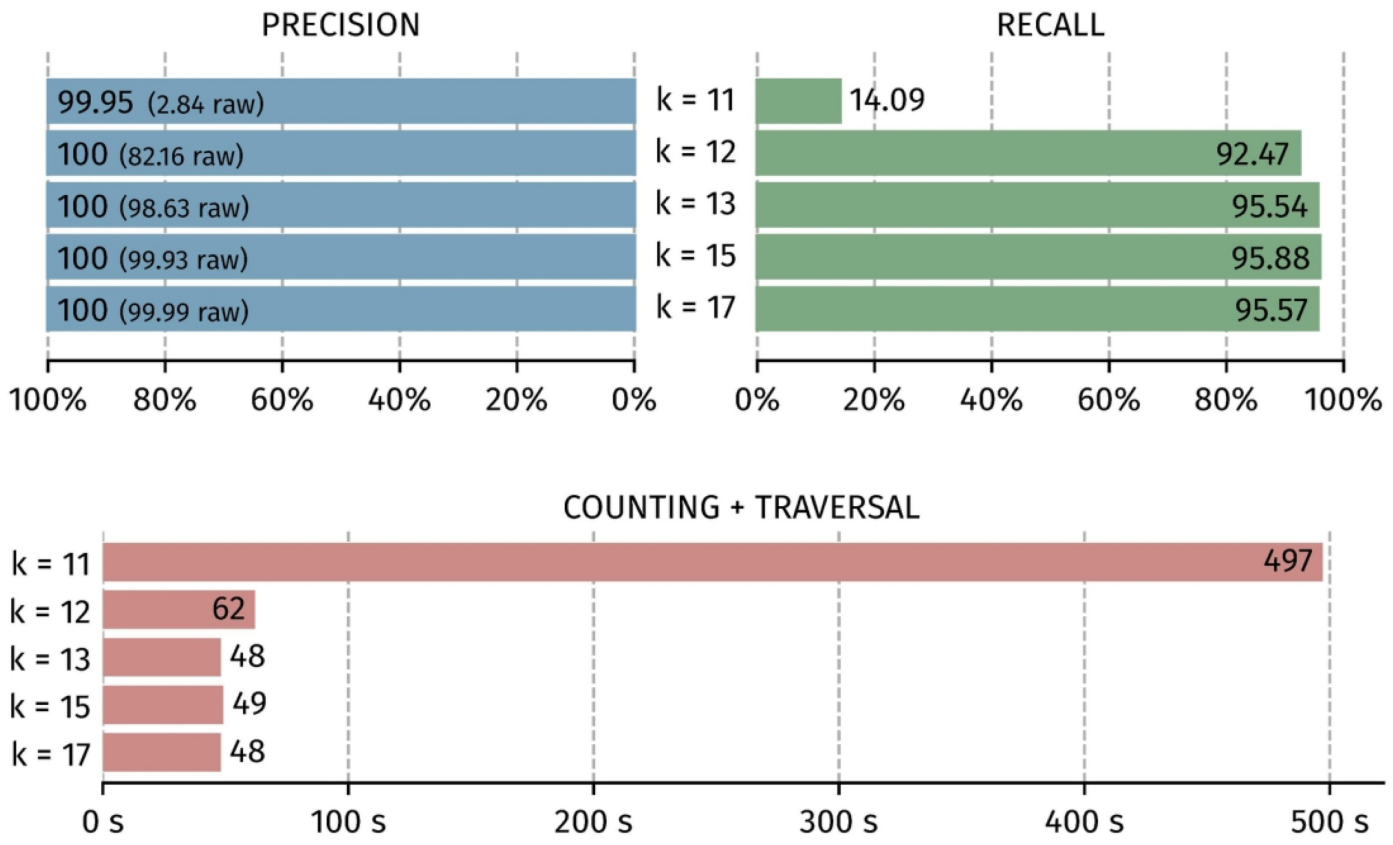
Impact of the k-mer size for precision, recall and consensus execution time.

The size of the k-mers (Fig. 7) has a strong impact. The minimum size giving acceptable results is 13 nt. Above this value, both precision and recall are good, and the computation time remains stable. Therefore, a default value of 15 seems appropriate.

**Figure 8.**
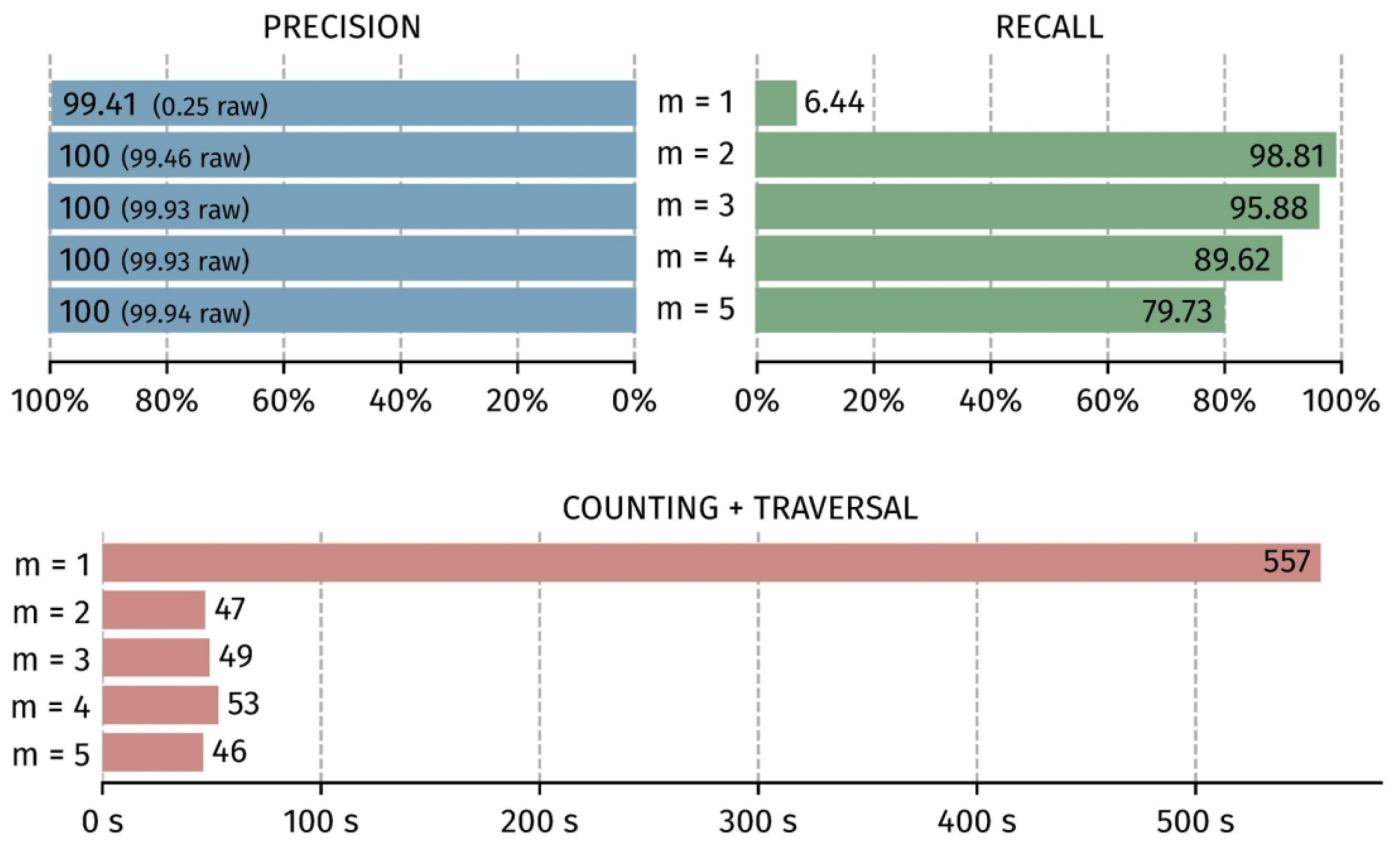
Impact of the k-mer abundance threshold for precision, recall and consensus execution time.

If the value of the low-abundance k-mer threshold (Fig. 8) is too low (i.e. set to 1), the de-Bruijn graphs generate many false positive sequences. This leads to poor precision and excessive computation time, the graph being too noisy and leading to reach the maximum number of traversal steps allowed by the software. On the other hand, the recall decreases as the threshold increases. A good balance is thus achieved with a trade-off value of 2 or 3 for an expected coverage of 30×.

### 4.5 Evaluation on real datasets

Finally, ConCluD was run and evaluated on real datasets, which were subsampled when required to ensure a maximum coverage of 30×. The results are presented in Table 3. We report only the raw precision, as the verification protocol is unavailable for most of the datasets. In the case of the Dunhuang datasets, this information is accessible and it gives a final precision of 100%. Recall remains high, except for the degraded sequences in Dunhuang-d56. In such cases, the molecular damage fragments the sequences and prevents ConCluD from performing consistent partitioning. The experiments conducted by the authors of DBGPS represent extreme conditions, and it is reasonable to assume a well-designed archival system would avoid such scenarios.

**Table 3.** Results of ConCluD on real datasets.

Dataset	Raw precision (%)	Recall (%)	Time (s)
Park et al.	99.87	99.99	0.67
Goldman et al.	71.66	97.99	4.93
Odeuropa	99.82	98.04	2.39
Dunhuang-d0	90.97	98.75	9.50
Dunhuang-d28	90.18	86.63	4.0
Dunhuang-d56	87.02	24.75	1.42
HorrorMovie	94.20	78.55	43.06

## 5 Conclusion

We presented a method based on de-Bruijn graphs to process sequencing reads derived from oligonucleotides and reconstruct the DNA sequences encoding a digital document. The originality and efficiency of our approach lie in a partitioning strategy that groups each sequence and all of its copies into the same subset. Each subset is then processed independently. This offers two key advantages. First, the resulting graphs are smaller, which simplifies their construction and handling. Second, this execution model allows for highly scalable processing. Our strategy is also compatible with longer oligonucleotide lengths, anticipating future technological advances that could enable the storage and retrieval of molecules larger than what is currently possible.

Our method does not require any prior knowledge of the payload’s structure. However, it does assume that each read contains a complete oligonucleotide, including its start and end primers. These primers are essential to initiate and terminate path-finding in the de-Bruijn graphs. Unfortunately, some Illumina paired-end sequencing protocols do not retain this information. The base calling step typically removes primers, as they are biologically irrelevant in that context. Nevertheless, the oligonucleotide can be reconstructed by merging read pairs and appending artificial primers. As such, the ConClud software is compatible with both long- and short-read sequencing technologies.

DNA-based data storage is a promising solution for long-term, high-density information archiving. In this context, recovering high-quality sequences after the sequencing step is of utmost importance, as it directly impacts the design of error-correcting codes tailored for DNA storage. When sequence reconstruction is highly accurate, the level of redundancy required for error correction can be significantly reduced, leading to substantial cost savings in the synthesis process. Moreover, being able to efficiently process large volumes of sequencing data is essential to ensure a smooth and scalable end-to-end data storage pipeline.

## Supplementary Material

btaf618_Supplementary_Data
