# Supplementary material for: De-Bruijn graph partitioning for scalable and accurate DNA storage processing
Source: Bioinformatics. 2025 Nov 9;41(11):btaf618. doi: 10.1093/bioinformatics/btaf618 (PMC12619639; doi:10.1093/bioinformatics/btaf618)
Supplement: btaf618_Supplementary_Data [file btaf618_supplementary_data.pdf]

De Bruijn Graph Partitioning for Scalable and Accurate DNA Storage Processing

Supplementary Materials

Florestan De Moor
IRISA-CNRS
Univ. Rennes, Inria
Rennes, France
florestan.de-moor@irisa.fr

Olivier Boullé
IRISA-CNRS
Univ. Rennes, Inria
Rennes, France
olivier.boulle@irisa.fr

Dominique Lavenier
IRISA-CNRS
Univ. Rennes, Inria
Rennes, France
lavenier@irisa.fr

November 4, 2025

1 Generation of the IM1, IM10 and IM100 synthetic datasets

The datasets used for performance evaluation have been constructed from datasets archived in the following Zenodo repository:

https://zenodo.org/records/15387164

6 datasets that represents DNA encoded JPEG images are available:

1. **IM1-140-2**: 1 pool of 31,294 oligonucleotides of size 140 nt. All oligonucleotides share the same pair of primers (2 primers).
2. **IM1-248-2**: 1 pool of 35,090 oligonucleotides of size 248 nt. All oligonucleotides share the same pair of primers (2 primers).
3. **IM10-140-20**: 10 pools of 36,583 oligonucleotides of size 140 nt. Each pool have a different pair of primers (20 primers).
4. **IM10-248-20**: 10 pools of 35,069 oligonucleotides of size 248 nt. Each pool have a different pair of primers (20 primers).
5. **IM10-248-11**: 10 pools of 35,069 oligonucleotides of size 248 nt. Each pool have the same start primer but a different end primer (11 primers).
6. **IM100-248-20**: 100 pools of 35,114 oligonucleotides of size 248 nt. Each pool have a different pair of primers among 10 start primers and 10 end primers (20 primers).

The structure of an oligonucleotide is as follows:

```
    20 nt      1 nt              98 nt or 206 nt              1 nt    20 nt
|START_PRIMER|BUFFER|--------------- PAYLOAD ---------------|BUFFER|END_PRIMER|
```

The length of the primers are 20 nt.

The length of the payload is 98 nt for oligos of size 140 nt and 206 nt for oligos of size 148 nt.

A 1-bit buffer is inserted between the payload and the primers due to biotechnological constraints. The payload contains encoded indexing and CRC information.

The scripts for generating the oligonucleotide datasets are available here:

`https://gitlab.inria.fr/pim/org.pim.dnarxiv/-/tree/main/paper_scripts/datasets/IM-1-10-100`

The generation of N pools of oligonucleotides from a binary file is performed as follows:

1. Split the binary file into N binary strings. N is determined by the target length of the oligonucleotides and the number of oligonucleotides by pool.
2. Apply a SHA256 hash to each binary strings with known key in order to obtain (pseudo) random binary strings of identical size, which is added to the binary string with an XOR operation. This operation ensures a good GC % balance and avoids large homopolymers.
3. Cut the binary strings into P fragments, relatively to the length of the oligonucleotides
4. Append a 16-bit index and 20-bit checksum to each fragment.
5. Convert fragments to DNA sequences with an encoding algorithm at a density of 1.66 bit/nt, ensuring a good GC balance and no homopolymeres.
6. Compute primers that do not hybridize with the fragments of a given pool.
7. Add primers and buffer to each fragment.

In order to be compliant with the DBGPS software, oligonucleotides from these datasets are modified. A 4 bytes index (= 16 bases) is inserted between the start primer and the payload, and a 2 bytes CRC (= 8 bases) is inserted between the payload and the end primer. The index is chosen to not have any homopolymers larger than 4 bases. The CRC is computed with the code of the public Python implementation of DBGPS. The helper scripts to convert the reference prints in the console the length and index start/end parameters to use to run ConCluD and DBGPS.

Long reads are generated with the PBSIM3 simulator, and short reads by the ART simulator.

As a result we get the following synthetic datasets:

- IM1-164ont2 made from IM1-140-2 and PBSIM3
- IM1-164ill2 made from IM1-140-2 and ART
- IM1-272ont2 made from IM1-248-2 and PBSIM3
- IM10-164ont20 made from IM10-140-20 and PBSIM3
- IM10-272ont20 made from IM10-248-20 and PBSIM3
- IM10-272ont11 made from IM10-248-11 and PBSIM3
- IM100-272ont20 made from IM100-248-20 and PBSIM3

2 Experiment Information

All the conditions and the implementation of the various experiments carried out within the framework of this article are available here:

`https://gitlab.inria.fr/pim/org.pim.dnarxiv/-/tree/main/paper_scripts`
